# A Randomised Control Study Comparing Ultrasonography with Standard Clinical Methods in Assessing Endotracheal Tube Tip Positioning

**DOI:** 10.2478/jccm-2024-0019

**Published:** 2024-04-30

**Authors:** Jayalekshmi Sreedevi, George Neethu, George Anjali, Paul Cherish

**Affiliations:** Travancore Medical College, Kollam, Kerala, India; Jubilee Mission Medical College and Research Institute, Thrissur, Kerala, India

**Keywords:** airway, ultrasound, endotracheal, tube tip position, cricoid cartilage, carina

## Abstract

**Introduction:**

Airway ultrasound has been increasingly used in correct positioning of endotracheal tube. We hypothesize that a safe distance between endotracheal tube tip and carina can be achieved with the aid of ultrasound.

**Aim of the study:**

Our primary objective was to determine whether ultrasound guided visualisation of proximal end of endotracheal tube cuff is better when compared to conventional method in optimal positioning of tube tip. The secondary objective was to find the optimal endotracheal tube position at the level of incisors in adult Indian population.

**Materials and Methods:**

There were 25 patients each in the conventional group and the ultrasound group. Conventional method includes auscultation and end tidal capnography. In the ultrasound group the upper end of the endotracheal tube cuff was positioned with an intent to provide 4 cm distance from the tube tip to the carina. X ray was used in both groups for confirmation of tip position and comparison between the two groups. Further repositioning of the tube was done if indicated and the mean length of the tube at incisors was then measured.

**Results:**

After x ray confirmation, endotracheal tube repositioning was required in 24% of patients in the USG group and 40 % of patients in the conventional group. However, this result was not found to be statistically significant (p = 0.364). The endotracheal tube length at the level of teeth was 19.4 ± 1.35 cm among females and 20.95 ± 1.37 cm among males.

**Conclusions:**

Ultrasonography is a reliable method to determine ETT position in the trachea. There was no statistically significant difference when compared to the conventional method. The average length of ETT at the level of incisors was 19.5 cm for females and 21 cm for males.

## Introduction

Endotracheal intubation is a lifesaving procedure that is routinely performed by anaesthesiologists, intensivists and emergency medicine physicians. Identifying the correct depth of endotracheal (ET) tube placement is important in preventing further pulmonary complications like endobronchial placement, accidental extubation etc.

Various methods have been used to confirm correct positioning of the ET tube tip.

Auscultation is often the easiest method, but it cannot decisively predict the distance between the tip of the ET tube and the carina. Confirmation by x-ray is associated with radiation exposure and may not be readily available. Though fibreoptic bronchoscopy is considered to be the gold standard, it requires technical skill and equipment availability.

Point of care ultrasound (USG) is being increasingly used in emergency medicine and intensive care units (ICU). Confirmation of ET tube position by ultrasonography can be a potential alternative to chest x-ray. Many studies have confirmed the usefulness of USG in early detection of accidental oesophageal intubation [1].

We hypothesize that by placing the upper edge of ETT cuff approximately 10 cm from sternal angle, or just below the cricoid cartilage if the first point is above the level of cricoid, it would correlate with position of ETT tip 4 cm above the carina. This study was done to evaluate the efficacy of ultrasound in assessing the correct placement of ET tube tip. The distance between the upper margin of cuff and tip of endotracheal tube was measured for the different sizes of ET tubes (Portex, Smiths medical, Minneapolis, USA) used in the study. It was 6 cm for ET tube sizes 7 to 8.5. The manubriosternal joint (sternal angle) is a reliable surface landmark for carina although there may be individual variations. The mean distance from endotracheal tube tip to carina that will prevent carinal impingement and endobronchial intubation can be taken as 4 cm [2]. The primary objective of this study was to determine whether ultrasound guided visualisation of proximal end of ET tube cuff is better when compared to conventional method in optimal positioning of ET tube tip. The secondary objective was to find the optimal ET tube position at the level of teeth in Indian adult population.

## Methods

This was a prospective randomized controlled trial with parallel group study design conducted in a multidisciplinary intensive care unit of a tertiary care hospital over a period of 6 months. Approval for the study was obtained from the institutional ethics committee (IEC Study Ref No: 62/21/IEC/JMMC&RI) and was registered under Clinical Trials Registry India (CTRI/2021/07/035029). All adult patients with a height more than 150 cm who required intubation with endotracheal tubes (ETT) of size 7 to 8.5 were included in the study. The exclusion criteria included pregnant patients, those who refused to give consent and those with abnormal airway, severe neck trauma or oropharyngeal pathology (hematoma, abscess, tumour etc).

A pilot study was conducted to observe the proportion of correct ETT placement. With an intended 95% confidence level and 90% power, a minimum sample size of 23 was calculated in each group. Patients were allotted to either the conventional group or intervention group (23 in each group) based on a computer-generated randomization chart. Random allocation was made in blocks in order to keep the sizes of groups similar. To accomplish that, a sample size of 50 was divided into 5 blocks (10 in each block). The study methodology did not permit blinding as we intervened and repositioned the endotracheal tube in both the conventional and USG group before the Xray if deemed indicated

Institutional ICU protocol was followed for intubation. It included rapid sequence intubation and confirmation of endotracheal tube placement using waveform capnography in both the groups.

In the conventional group, the investigator positioned the ET tube based on the “guiding black mark” at the level of vocal cords or at 22 cm length at the level of incisor teeth in males and 20 cm in females. Auscultation was done to rule out endobronchial intubation.

**Fig. 1.**
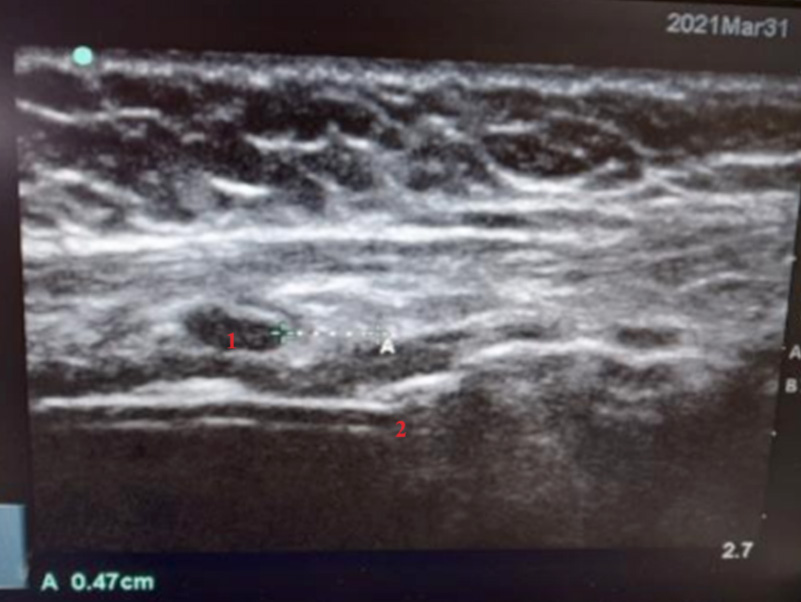
Ultrasound image showing cricoid cartilage (1) and cutoff point (2)

**Fig. 2.**
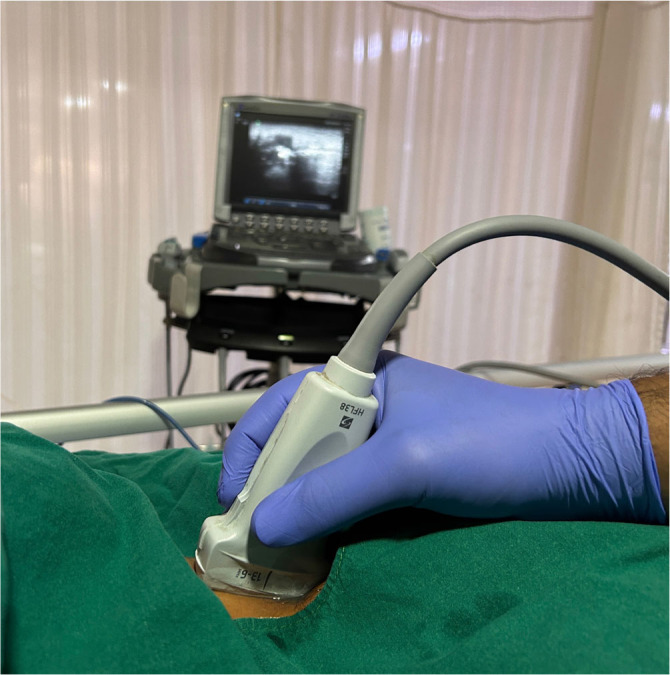
Alignment of the linear USG probe for airway imaging

In the interventional group, sternal angle was identified by the investigator experienced in airway sonography using the linear transducer (13-6 MHz) probe of ultrasound (Sonosite M – turbo, Washington, USA). The same was marked on the skin using a surgical marker (Romsons, Denmark) and another point P1 was noted 10 cm cephalad to the sternal angle using a sterile surgical ruler (Romsons, Denmark). Lower end of cricoid cartilage was also identified by ultrasonography and marked as point P2. The proximal end of ETT cuff was identified by abrupt cut off of acoustic shadow of ETT (TUBE CUT OFF POINT) and noted as point P3. The straight-line acoustic image of ETT was replaced by a shredded wavy line created by the mucosal- cuff interface as shown in Figure 1. In Figure 2 we can see the midline alignment of the linear USG probe in the neck to get the right image.

**Fig. 3.**
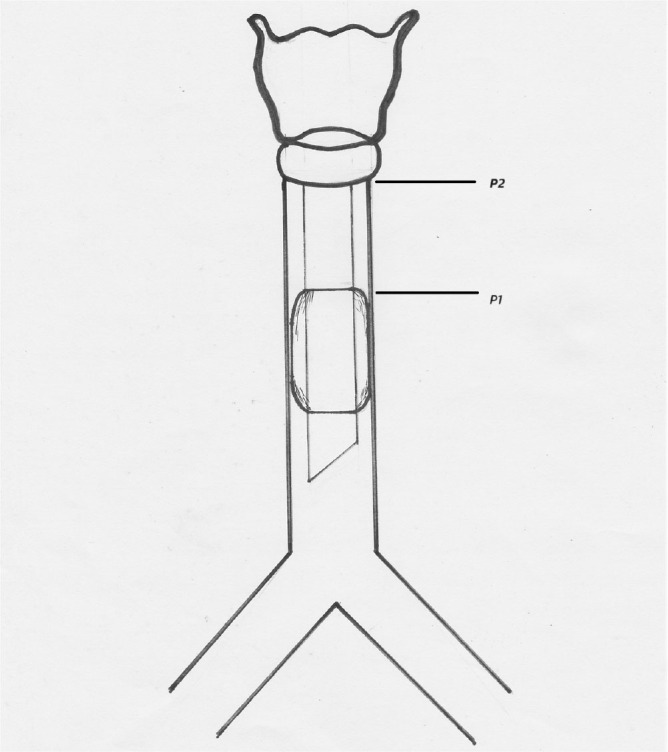
Schematic diagram of the trachea with ETT in situ

The investigator adjusted the upper end of the cuff to the point P1 (10 cm above sternal angle) or just below the level of cricoid cartilage (P2) whichever was lower. If P2 was below P1, the upper end of the ETT cuff was positioned at P2. If the lower end of cricoid cartilage was above P1, the upper end of the ETT cuff was positioned at P1. The below Figure 3 illustrates the abovementioned points and landmarks.

First step after intubation in the interventional group was to readjust the proximal end of the ETT cuff by direct visualization using ultrasound. By keeping the upper end of the ETT cuff in the midline of the linear transducer probe the “tube cut off point” was marked on the skin. The distance between the “tube cut off point” and P1 or P2 as indicated, was measured using the sterile surgical ruler and the ETT was inserted or withdrawn accordingly.

The position of endotracheal tube tip was reconfirmed using chest X-ray AP view with the neck in neutral position. In case any complications occurred before X-ray confirmation, (saturation fall below 90%, increase in peak airway pressures, audible leak etc), the plan was to rule out endobronchial intubation or endotracheal tube dislodgement by auscultation. The ET tube would then be adjusted and positioned according to clinical judgement and the adjusted length noted.

Distance between ET tube tip and carina was measured on the chest Xray using distance annotation tool. The optimal position of ETT tip on X-ray was taken as 3 – 5 cm above carina to determine the appropriateness of the tube position. If the ETT tip position was found to be inappropriate, then the ETT was repositioned. The length of ETT at the level of incisor teeth was measured and recorded in all the three stages i.e. immediately after intubation, after ultrasound guided positioning and after taking chest x ray.

## Results

A total of 50 study participants were recruited during the study period. All of them were included in the final statistical analysis of which 25 study participants were in the interventional (USG group) and 25 study participants were in the conventional group. Both the groups were matched for age, gender, height and size of the endotracheal tube (Table 1).

**Table 1.** Demographic data

**Parameter**	**USG group**	**Conventional group**	**p value**
Age (years)	52.48 ± 17.65	50.04 ± 16.61	0.617
Height (cm)	166. ± 18.9	162.7 ± 6.9	0.140
ETT size (mm)	7.9 ± 0.45	7.6 ± 0.54	0.055
Female No: (%)	5 (20%)	12 (48%)	
Male No: (%)	20 (80%)	13 952%)	0.072

In the USG group, the lower end of cricoid cartilage (P2) was seen below or at the level of point P1 in 5 (20%) patients and hence the upper end of endotracheal tube cuff was positioned at P2. The “tube cut off point” was aligned to P1 in the remaining 20 (80 %) patients. Repositioning of the endotracheal tube was done after USG assessment in 14 (56%) patients in this group. None of these patients had any complications prior to x-ray confirmation that required switching over to the auscultation method. The mean distance from the lower end of cricoid cartilage to suprasternal notch was 4.64 ± 1.02 cm. The average length of manubrium sterni was 5.58 ± 0.58 cm. The mean time taken for USG guided positioning was 13.4 ± 6.8 minutes.

In the conventional group also, no patients developed any complications (desaturation, high airway pressures or leak) prior to x ray confirmation.

After x ray confirmation, endotracheal tube repositioning was required in 6 patients (24%) in the USG group and 10 patients (40%) in the conventional group. However, this result was analysed using Chi square tests and was not found to be statistically significant (p = 0.364).

Among the 20 patients whose endotracheal tube cuff was positioned at P1 in the USG group, 4 patients required repositioning based on x ray. Whereas among the 5 patients in the USG group whose endotracheal tube cuff was positioned at the lower end of cricoid cartilage, 2 patients required repositioning after X-ray. This was also not found to be statistically significant by Chi square tests (p = 0.343). The endotracheal tube length at the level of teeth was 19.4 ± 1.35 cm among females and 20.95 ± 1.37 cm among males. The ETT length at teeth for patients with various heights were as given in Table 2

**Table 2.** Length of ETT at incisors varying with height

	**Height(cm)**	**Mean (± SD)**
ETT length at teeth after x ray	<165	20.01(1.35)
	165–180	20.73 (1.26)
	>180	23.5 (0.50)

## Discussion

Ultrasonography is being increasingly used in various specialties like anaesthesia, emergency medicine and critical care. Its use in regional anaesthesia and focused intensive care echocardiography is now a standard practice. Its importance in point of care lung ultrasound in critical care is known especially since the Covid era [3]. Naturally intensivists got used to airway ultrasonography and its possibilities. But the research on airway ultrasonography was mainly related to intubation confirmation, but not the position of the ETT [4,5,6]. There were proposals on the position identification based on indirect methods like presence of lung sliding in both lungs [7]. The presence of bilateral lung slide can be as good as capnography in avoiding oesophageal intubation [8,9].

Our study was done to evaluate the efficacy of ultrasound in assessing the position of endotracheal tube in ICU patients. However, we could not find a statistically significant advantage in using USG over the conventional method. Probably we should conduct similar study in a larger group before confirming that USG does not change the outcome in ETT positioning. The upper end of the ETT cuff is easy to identify although only a few studies have considered it [10].

We feel that our skill in using USG for ETT positioning has improved over time so that we use USG as a routine to assess the airway after intubation. However, we do admit that the method we used in the USG group to confirm the positioning is time consuming though less compared to x ray. To use it as a routine, the airway USG skill of the doctors need to be improved. In the USG group, the average endotracheal tube length at the level of teeth was found to be 21± 0.68 cm for males and 19± 1.38 cm for females. The average distance from the upper end of endotracheal tube cuff to the lower end of cricoid cartilage was found to be 0.5± 0.22 cm for males and 0.7± 0.35 cm for females. Hence if we could position the upper end of the endotracheal tube cuff just below the lower end of cricoid cartilage in all the study participants in the USG group, the average endotracheal tube length at the level of teeth would be around 20.5 cm for males and 18.5 cm for females. The methodology we used in our study to confirm the endotracheal tube position was time consuming though less when compared to Xray. We can easily measure the distance between the upper end of endotracheal tube cuff and lower end of cricoid cartilage and position the upper end of endotracheal tube cuff to just below the lower end of cricoid cartilage. This method would be much less time consuming and may also provide safe margin to prevent accidental extubation. Further research should be conducted to look for its generalisability; but we consider it as a practical and possible option.

Other limitation of our study was the lack of blinding of the doctors doing the intubation and positioning. For this, we did consider the possibility of doing the conventional assessment and USG assessment in both the groups. But if not in position, repositioning after the conventional or USG assessment would then not be a possibility. Hence, we deferred blinding of the airway assessors.

Other option used by researchers to identify the position of the ETT within the trachea was to determine the distance from the vocal cords to the upper end of the saline filled cuff. In a study involving 105 patients, USG was reliably used to locate the saline inflated cuff and the vocal cords [11]. It seems to be a reliable possibility, but needs the vocal cords to be identified prior to intubation which might be practically difficult. Also, the chance of bringing this to routine use is challenging since saline filled cuff is not a universal practice. We usually do not prefer the cuff to be at the level of cricoid and so lower level of cricoid cartilage might be a better reference point than the vocal cords. But saline filled cuff has the potential advantage of reducing the learning curve of USG guided ET tube positioning [12].

Airway ultrasonography has been successfully used in determining the appropriately sized ET tube in paediatric population. In that study, presence of bilateral lung sliding was used to confirm the correct tracheal position of ETT [13]. It is probably difficult to find a formula for paediatric population for correct placement of ETT by USG. We rather might have to depend on the distance from airway structures like vocal cord or cricoid cartilage. In neonates, the distance from the ETT tip to superior portion of the right pulmonary artery (the anatomical equivalent of the carina) was used for USG positioning of ETT. They found it equally accurate to chest Xray in determining the ETT position [14].

USG might have an advantage over the conventional method when it comes to other confounding factors like obesity. It could increase the accuracy and sensitivity in detecting the correct placement of ETT when compared to the auscultation method [15].

In ICU the ETT position is likely to change during therapeutic patient position changes or during nursing care. It is not practical to repeat the chest x ray each time and USG might be an option in such scenarios [16].

Successful usage of USG in airway evaluation and management has been comprehensively reviewed [17]. USG was found reliable in predicting difficult airway, post extubation laryngeal oedema, cricothyrotomy guidance and ETT confirmation.

The practice of a fixed position of ETT at the incisors for males and females varies in different parts of the world. Though the concept is right, it does not seem to be on the basis of sound anatomical evidence. On confirmed position after chest Xray, we found the average length to be around 19.5 cm for females and 21 cm for males. A similar assessment in Korean population found this to be around 20 cm for females and 22 cm for males [18]. The vocal cord – carina distance can vary. So, deciding on a fixed length at lip or teeth can lead to inappropriate positioning [19]. So, it is imperative that sound ETT positioning methods are in place to prevent inadvertent events especially in critical care units.

With more awareness in the field and increasing practice of airway ultrasound, a quick and reliable technique will surely come up [20].

## Conclusion

Ultrasonography is a reliable method to determine ETT position in the trachea. But we could not find any statistically significant difference when compared to the conventional method. The average length of ETT at the level of incisors was 19.5 cm for females and 21 cm for males.
